# Validating patient and physician versions of the shared decision making questionnaire in oncology setting

**DOI:** 10.15171/hpp.2019.15

**Published:** 2019-05-25

**Authors:** Babak Nejati, Chien-Chin Lin, Vida Imani, Maria Browall, Chung-Ying Lin, Anders Broström, Amir H Pakpour

**Affiliations:** ^1^Hematology and Oncology Research Center, Tabriz University of Medical Sciences, Tabriz, Iran; ^2^Department of Laboratory Medicine and Division of Hematology, Department of Internal Medicine, National Taiwan University Hospital, Taipei, Taiwan; ^3^Graduate Institute of Clinical Medicine, National Taiwan University, Taipei, Taiwan; ^4^Pediatric Health Research Center, Tabriz University of Medical Sciences, Tabriz, Iran; ^5^Department of Nursing, School of Health and Welfare, Jönköping University, Jönköping, Sweden; ^6^Department of Rehabilitation Sciences, The Hong Kong Polytechnic University, Hung Hom, Hong Kong; ^7^Social Determinants of Health Research Center, Qazvin University of Medical Sciences, Qazvin, Iran

**Keywords:** Confirmatory factor analysis, Cancer, Rasch, Instrumental study

## Abstract

**Background:** This study investigated the psychometric properties of the 9-Item Shared Decision-Making Questionnaire (SDM-Q-9) and the 9-Item Shared Decision-Making Questionnaire–Physician version (SDM-Q-Doc) using comprehensive and thorough psychometric methods in an oncology setting.

** Methods: ** Cancer survivors (n=1783; 928 [52.05%] males) and physicians (n=154; 121[78.58%] males) participated in this study. Each cancer survivor completed the SDM-Q-9. Physicians completed the SDM-Q-Doc for each of their cancer patient. Confirmatory factor analysis (CFA) and Rasch model were used to test the psychometric properties of SDM-Q-9 and SDM-Q-Doc.

**Results:** SDM-Q-9 and SDM-Q-Doc demonstrated unidimensional structure in CFA and Rasch model. In addition, the measurement invariance was supported for both SDM-Q-9 and SDM-QDoc across sex using the multigroup CFA. Rash analysis indicates no differential item functioning(DIF)across sex for all the SDM-Q-9 and SDM-Q-Doc items. SDM-Q-9 and SDM-Q-Doc were moderately correlated (r=0.41; P<0.001).

**Conclusion: ** SDM-Q-9 and SDM-Q-Doc are valid instruments to assess shared decision making in the oncology setting.

## Introduction

Cancer is one of the most important causes of death and human health issues during the past decades.^1^ According to the World Health Organization (WHO) report, cancer accounted for near 9.6 million deaths in 2018 globally.^2^ The most common cancer types include lung, breast, prostate, and gastrointestinal tract cancers. With the development of advanced diagnostic tools and the progress made in various cancer treatments, the survival rates of cancer patients have kept increasing. In addition to the medical parts, efforts have been made to improve the quality of their physical and psychosocial support during the past few years.^1^ However, many of the cancer survivors still experience several unmet needs, including education, information, communication and provider relationship.^3^

In order to achieve the quality of cancer care, healthcare providers should have good communication with cancer survivors. Indeed, it is claimed that the optimal decision for a patient can be made using the model of shared decision making; that is, at least a clinician and a patient share information to each other. Clinicians offer treatment options with potential risks and benefits; patients express preferences and values.^4^ Through shared decision making, active participation in the decision-making process is increased with the following benefits: improved knowledge for patient, decreased decision conflict between clinicians and patients, and facilitated decision reaching.^5^ Additionally, less anxiety and better treatment outcomes have been proposed to those patients who used shared decision making.^6^ The benefits of shared decision making are also been proposed for cancer survivors that oncological decisions made by shared decision making can enrich the treatment process for patients to gain benefits from curative cancer treatments.^7^

Thus, the 9-item Shared Decision Making Questionnaire (SDM-Q-9) and the Shared Decision Making Questionnaire-physician version (SDM-Q-Doc) have been developed recently.^8-10^ Both measures were found to be feasible and effective in interventions.^11^ However, psychometric properties of both SDM-Q-9 and SDM-Q-Doc need additional investigation because of the following reasons. Specifically, to the best of our knowledge, only two studies have validated the SDM-Q-9 using a sample of breast cancer patients or a sample majority consisted of colon, breast, and stomach cancer patients.^12,13^ Another study has validated the SDM-Q-Doc using a sample of medical oncologists who rated SDM-Q-Doc for their oncology patients with majority diagnoses were colon cancer (40.4%) and breast cancer (35.2%).^7^ Thus, psychometric evidence of SDM-Q-9 and SDM-Q-Doc is understudied in the oncology setting. For example, we are unsure whether SDM-Q-9 is sound and valid for cancer survivors other than the aforementioned cancers; we have no information whether SDM-Q-Doc is reliable and can be applied to cancer survivors when the physician raters are not oncologists. Given that the scientific nature is to accumulate evidence using different methods,^14^ especially that the psychometric properties are highly depended on the studied samples,^15^ further psychometric evaluation for SDM-Q-9 and SDM-Q-Doc is thus crucial. Specifically, SDM-Q-9 should be tested among different types of cancer diagnoses and SDM-Q-Doc should be examined among various areas of physician expertise.

In addition, to our knowledge, both SDM-Q-9 and SDM-Q-Doc have not been investigated for their measurement invariance and differential item functioning (DIF) across sex. Given that males and females usually have different thoughts and diverse interpretations in item descriptions,^16^ it is essential for healthcare professionals to understand whether male and female cancer survivors interpret the SDM-Q-9 items similarly; whether male and female physicians interpret the SDM-Q-Doc items similarly. After ensuring that the measures are measurement invariant and have no DIF items across sex, healthcare professionals can conclude that males and females report the same thing in their shared decision making using SDM-Q-9 and SDM-Q-Doc. In other words, we need not to worry about the gender bias when using either SDM-Q-9 or SDM-Q-Doc.

The purpose of this study was to investigate the psychometric properties of SDM-Q-9 and SDM-Q-Doc using comprehensive and thorough psychometric methods in an oncology setting.

## Materials and Methods

### Participants and recruitment process

The sample of this methodological study was recruited from five teaching hospitals in cities of Tehran, Tabriz, Qazvin and Karaj from November 2017 to July 2018. Participants included cancer survivors and physicians. Patients were included in this study if they met the following criteria: (1) 18 years or more, (2) confirmed diagnosis of cancer by pathological examination, and (3) the ability to communicate in Persian, and providing informed consent. Exclusion criteria were: (1) severe cognitive impairment as suggested by mini mental state examination (MMSE) score; i.e., less than 21, (2) with visual impairment, and (3) with major psychiatric disorders (as diagnosed by a psychiatrist).

The patients were informed the study by their treating physicians (i.e., the gynecologist, oncologist, and urologist who agreed to participate in this study) and each physician invited 12 of their outpatients after giving the consultation. However, the patients were well informed and ensured that there will be no changes in their treatment if they reject to participate. If a patient agreed to participate in this study and signed a written informed consent, the patient, his/her physician, and one of his/her family member completed questionnaires described in the *Measures* section.

### Translation procedure for SDM-Q-9 and SDM-Q-Doc

Given that SDM-Q-9 and SDM-Q-Doc are paralleled instruments completed by different parties (i.e., patients and physicians), we considered that translating both instruments simultaneously could tackle the characteristics of the paralleled instruments. Several steps were taken to translate both measures (i.e., SDM-Q-9 and SDM-Q-Doc) from English to Persian based on international guidelines on cross-cultural adoption of self-report measures.^17-19^ In the first stage, two bilingual translators who were native Persian speakers independently translated both SDM-Q-9 and SDM-Q-Doc from English to Persian. In the next step, the translators and a recording observer compared the two translations. Any discrepancies between the translations were identified and resolved to synthesize as an interim Persian version. In the third stage,‏ the synthesized Persian measures were translated back into English by two bilingual translators who were blinded to the English versions. An expert committee (including oncologist, psychiatrist, methodologist, nurse, psychologist, psychometrist and the translators) was convened to consolidate all the translated versions of the questionnaires and meanwhile tackle cultural adaptation. Necessary changes were made after reviewing all documents to achieve linguistic equivalence between the Persian and English versions. The pre-final Persian versions were then piloted on patients (mean age = 57.6 years, 23 males and 24 females) and physicians (mean age = 35.2 years, 14 males and 10 females) to make sure that the Persian versions were equivalent to their English correspondences in clinical setting. The final Persian SDM-Q-9 was administrated on 1783 cancer patients and Persian SDM-Q-Doc on 154 physicians.

### Measures

The physician completed SDM-Q-Doc and a questionnaire on physician satisfaction for that patient. One family member nominated by the patient (e.g., spouse, parent, child, or sibling) completed the Family Decision-Making Self-Efficacy Scale. A research assistant contacted the patient to complete a series of questionnaires (please see the *followings* for detailed information) and background information sheet.

*9-Item Shared Decision-Making Questionnaire (SDM-Q-9)*

The SDM-Q-9 consists of 9 items and evaluates how a patient perceives his or her involvement in the shared decision making during the medication consultation.^9^All the items were rated on a 6-point Likert scale from 0 (completely disagree) to 5 (completely agree), where a higher score indicates higher involvement of a patient in decision making. The single-factor structure of the SDM-Q-9 has been verified in both confirmatory factor analysis (CFA) and Rasch model.^12,20^ The internal consistency of the SDM-Q-9 in this current study was 0.919.

*9-Item Shared Decision-Making Questionnaire–Physician version (SDM-Q-Doc)*

The SDM-Q-Doc has parallel nine items to the SDM-Q-9 with different subject terms in each item (the subject term is *my doctor* in SDM-Q-9 and *I* in SDM-Q-Doc). All the items were rated on a 6-point Likert scale from 0 (completely disagree) to 5 (completely agree), where a higher score indicates higher involvement of a patient in decision making.^10^ The single-factor structure of the SDM-Q-9 has been verified in the CFA,^21^ and the SDM-Q-Doc has its linguistic validity established in Persian.^18^ The internal consistency of the SDM-Q-Doc in this current study was 0.820.

*Hospital Anxiety and Depression Scale (HADS)*

The HADS consists of 14 items assessing depression (7 items) and anxiety (7 items). All the items were rated on a four-point Likert scale from 0 to 3, where a higher score indicates more depression or anxiety. Moreover, the Persian version of the HADS has been validated in a sample with epilepsy.^22^ The internal consistency of the depression and anxiety domains in this current study were 0.83 and 0.88.

*Family Decision-Making Self-Efficacy Scale (FDMSE)*

The FDMSE consists two 13-item scenarios to assess family self-efficacy in decision making.^23^ Sample items in the two scenarios are “*If my loved one prefers to have help in making health care decisions, I am confident that I will be able to help make decisions about his/her health care* (Conscious scenario).” and “*If my loved one becomes too ill to make health care decisions, I am confident that I will be able to make decisions about his/her health care* (Unconscious scenario)”. Because we were interested in the process of making the decision by patients and all our participants had capacity in decision making, we only used the conscious scenario and did not use the unconscious scenario. Given that there is no Persian version of the FDMSE, we translated the FDMSE into Persian using standard procedure to ensure its linguistic validity. The internal consistency of the FDMSE in this current study was 0.82.

*Patient Self-Advocacy Scale (PSAS)*

The PSAS consists of 12 items assessing illness education (4 items), assertiveness (4 items), and mindful non-adherence (4 items). All the items were rated on a 5-point Likert scale from 1 to 5, where a higher score indicates better illness literacy, higher confidence in taking care of illness, and paying more attention to non-adherence. Moreover, the Persian version of the PSAS has been validated in a sample with chronic illness.^24^ The internal consistency of the PSAS in this current study was 0.85.

*Insomnia Severity Index (ISI)*

The ISI consists of 7 items assessing the severity of sleep problem with all items rated on a 5-point Likert scale from 0 to 4, where a higher score indicates severe insomnia. Moreover, the Persian version of the ISI has been validated in a sample with sleep problems.^25^ The internal consistency of the ISI in this current study was 0.89.

*Epworth Sleepiness Scale (ESS)*

The ESS consists of 8 items assessing the severity of daytime sleepiness with all items rated on a 4-point Likert scale from 0 to 3, where a higher score indicates greater sleepiness. Moreover, the Persian version of the ESS has been validated in Iranian children and adolescents.^26^ The internal consistency of the ESS in this current study was 0.82.

*Physician satisfaction*

Four items were designed in this study to examine the physician satisfaction. The four items asked to what extent the physician is satisfied with (1) the information provided about the disease; (2) the risk of recurrence; (3) the side effects of treatment; and (4) the time dedicated to informing the patient. All items were rated from 0 to 8, with a higher score indicates the higher level of physicians’ satisfaction with the provided information. The internal consistency of the four items in this current study was 0.88.

*7-item Short Assessment of Patient Satisfaction (SAPS)*

The SAPS consists of 7 items assessing the patient satisfaction with all items rated on a 5-point Likert scale from 0 to 4, where a higher score indicates higher level of satisfaction.^27^ Given that there is no Persian version of the SAPS, we translated the SAPS into Persian using standard procedure to ensure its linguistic validity. The internal consistency of the SAPS in this current study was 0.80.

### Data analysis

Two series of psychometric testing were conducted with one using classical test theory and another using Rasch analysis. The classical test theory analyses were done using IBM SPSS 23.0 (IBM Corp., Armonk, NY) or Mplus (version 7.4; Los Angeles, CA); Rasch analyses were done using Winstep 4.1.0 software (winsteps.com, Beaverton, OR).

For the testing using classical test theory, we used CFA, internal consistency using Cronbach’s α, corrected item-total correlation, test-retest reliability using Pearson correlation, acceptance (i.e., the completion rate for each item), ceiling and floor effects (i.e., how many percentages of the participants have the lowest or highest score in each instrument), and standard error of measurement. Moreover, both SDM-Q-9 and SDM-Q-Doc were tested as having a single-factor structure each in the CFA, and the CFA results were used to report factor loadings, average variance extracted, and composite reliability. Several cutoffs were then used to determine the acceptable properties. For CFA, a nonsignificant χ^2^ test, a comparative fit index (CFI) >0.9, a Tucker-Lewis index (TLI) >0.9, a root mean square error of approximation (RMSEA) <0.08, and a standardized root mean square residual (SRMR) <0.08 support the single-factor structure for SDM-Q-9 or SDM-Q-Doc.^28^ For internal consistency, Cronbach’s α is expected to be higher than 0.7^29^; for corrected item-total correlation and test-retest reliability, values are expected to be higher than 0.4.^28^ A higher acceptance and a lower standard error of measurement indicate better psychometric properties.^29^ Moreover, factor loading higher than 0.4; average variance extracted higher than 0.5, and composite reliability higher than 0.6 are in anticipation.^29^

Three nested CFA models were additionally used to test the measurement invariance of both instruments (viz., SDM-Q-9 and SDM-Q-Doc) across males and females. The three models were (1) a configural model that treats SDM-Q-9 and SDM-Q-Doc as a single-factor structure, (2) a model that constrains males and females having equal factor loadings in the single-factor structure, and (3) a model that constrains males and females having equal factor loadings and equal item intercepts in the single-factor structure. A χ^2^ difference test, ∆CFI, ∆SRMR, and ∆RMSEA were used to examine whether measurement invariance is supported. If the χ^2^ difference test is nonsignificant, the measurement invariance is supported. If the χ^2^ difference test is significant, we expected to have ∆CFI, ∆SRMR, and ∆RMSEA all less than 0.01 to support the measurement invariance.^30^

For the testing using Rasch analysis, each item was examined whether it contributes sufficiently and appropriately to the underlying single-factor in shared decision making. Specifically, both infit mean square (MnSq) and outfit MnSQ range between 0.5 and 1.5 are expected, where a value <0.5 indicates the redundancy of an item and a value >1.5 indicates the inappropriateness of an item.^29^ In addition, difficulty of each item was calculated using the Rasch model with an additive unit (viz., *logit*). Given the nature of sample-free in Rasch (i.e., the psychometric results derived from the Rasch are not influenced by the sample characteristics), item separation reliability, person separation reliability, item separation index, and person separation index were demonstrated. The acceptable value for separation reliability was higher than 0.7; for separation index was higher than 2.^31^ DIF was then computed to examine whether males and females interpret the item description similarly and a DIF contrast (i.e., item difficulty for females- item difficulty for males) >0.5 indicates substantial DIF.^32^

After ensuring the psychometric properties of both SDM-Q-9 and SDM-Q-Doc, we investigated the Pearson correlations between the two instruments. Then, Pearson correlation was applied again between SDM-Q-9 and the relevant and external criteria as follows, depression, anxiety, family decision making self-efficacy, illness education, assertiveness, mindful non-adherence, insomnia, sleepiness, physician satisfaction, and patient satisfaction. Pearson correlation was applied again between SDM-Q-Doc and the followings, depression, anxiety, insomnia, sleepiness, physician satisfaction, and patient satisfaction.

## Results

### Participants’ characteristics

The M (SD) age of the 1783 cancer survivors was 58.63 (12.83) years, and slightly more than half of them were males (n = 928; 52.05%). In average, their educational years was 6.03 (2.96), time since diagnosis was 2.87 (1.66) years, MMSE score was 25.12 (1.90), and Karnofsky Performance Scale Score was 70.68 (12.81). More than half of the cancer survivors were currently married (n = 1198; 67.19%). Regarding their diagnosis, the highest three were gastrointestinal cancer (n = 582; 32.64%), breast cancer (n = 284; 15.92%), and skin cancer (n = 264; 14.81%). In terms of the characteristics of 154 physicians, their mean age was 38.69 (10.34) years with more than three quarters were males (n = 121; 78.58%). In average, they have been employed as a physician for 9.62 (5.81) years. Additionally, most of them were gynecologist (n = 63; 40.91%), followed by oncologist (n = 34; 22.08%), and urologist (n = 57; 37.01%; Table 1).

### Psychometric properties of the SDM-Q-9

The SDM-Q-9 was found to have promising psychometric properties in both item and scale levels. In the item level, acceptances were high (96.7% to 99.3%); factor loadings derived from CFA were satisfactory (0.77 to 0.94); corrected item-total correlations were strong (0.62 to 0.77); test-retest reliability calculated using Pearson correlation was excellent (0.70 to 0.93); infit (0.85 to 1.23) and outfit MnSq (0.83 to 1.12) derived from Rasch analysis were fit; and no substantial DIF (DIF contrast = -0.22 to 0.28) was found across sex (Table 2).

In the scale level tested using classical test theory, the SDM-Q-9 had low ceiling (2.13%) and floor effects (3.87%); high internal consistency (α = 0.919); satisfactory fit indices except for significant χ^2^ test in CFA (CFI = 0.971, TLI = 0.944, RMSEA = 0.070, and SRMR = 0.050); acceptable average variance extracted (0.70) and composite reliability (0.95); low standard error of measurement (2.89); acceptable test-retest reliability (*r* = 0.85; Table 3); supported measurement invariance (nonsignificant χ^2^ tests between the nested models; Table 4). The Rasch analysis also supported the promising psychometric properties of the SDM-Q-9 in scale level: separation reliability (0.84 and 0.92) and separation indices (3.47 and 2.28) were adequate (Table 3).

### Psychometric properties of the SDM-Q-Doc

The SDM-Q-Doc was found to have promising psychometric properties in both item and scale levels. In the item level, acceptances were high (97.3% to 99.3%); factor loadings derived from CFA were satisfactory (0.60 to 0.88); corrected item-total correlations were strong (0.41 to 0.73); test-retest reliability calculated using Pearson correlation was excellent (0.70 to 0.86); infit (0.80 to 1.16) and outfit MnSq (0.85 to 1.15) derived from Rasch analysis were fit; and no substantial DIF (DIF contrast = -0.39 to 0.43) was found across sex (Table 2).

In the scale level tested using classical test theory, the SDM-Q-9 had low ceiling (5.19%) and floor effects (7.80%); high internal consistency (α = 0.820); satisfactory fit indices except for significant χ^2^ test in CFA (CFI = 0.968, TLI = 0.939, RMSEA = 0.078, and SRMR = 0.052); acceptable average variance extracted (0.52) and composite reliability (0.91); low standard error of measurement (2.64); acceptable test-retest reliability (*r* = 0.81; Table 3); supported measurement invariance (nonsignificant χ^2^ tests between the nested models; Table 4). The Rasch analysis also supported the promising psychometric properties of the SDM-Q-9 in scale level: separation reliability (0.79 and 0.84) and separation indices (2.31 and 2.92) were adequate (Table 3).

### Correlations between SDM-Q-9, SDM-Q-Doc, and external criteria

A moderate and positive correlation between SDM-Q-9 and SDM-Q-Doc (*r* = 0.41; *P *< 0.001) was found (Table 5). Additionally, the SDM-Q-Doc was significantly correlated with depression (*r* = -0.60; *P *< 0.001), anxiety (*r* = -0.66; *P *< 0.001), family decision-making self-efficacy (*r* = 0.52; *P *< 0.001), illness education (*r* = 0.53; *P *< 0.001), assertiveness (*r* = 0.62; *P *< 0.001), mindful non-adherence (*r* = 0.40; *P *< 0.001), insomnia (*r* = -0.58; *P *< 0.001), sleepiness (*r* = -0.60; *P *< 0.001), physician satisfaction (*r* = 0.41; *P *< 0.001), and patient satisfaction (*r* = 0.69; *P *< 0.001). The SDM-Q-9 was significantly correlated with depression (*r* = -0.55; *P *< 0.001), anxiety (*r* = -0.62; *P *< 0.001), insomnia (*r* = -0.49; *P *< 0.001), sleepiness (*r* = -0.53; *P *< 0.001), physician satisfaction (*r* = 0.67), and patient satisfaction (*r* = 0.33; *P *< 0.001).

## Discussion

This study, to the best of our knowledge, is the first to examine the measurement invariance and DIF across sex for both SDM-Q-9 and SDM-Q-Doc in different diagnoses of cancer survivors, including gastrointestinal cancer, breast cancer, multiple myeloma, skin cancer, lung cancer, and genitourinary cancer. In addition, comprehensive and thorough evidence in their psychometric properties was examined. Specifically, the unidimensionality was verified by our findings from CFA and Rasch models for both SDM-Q-9 and SDM-Q-Doc. Our DIF and measurement invariance findings further suggested that male cancer survivors did not have different interpretation in the SDM-Q-9 items from female cancer survivors; the male physicians did not interpret the SDM-Q-Doc items differently from female physicians. Moreover, both SDM-Q-9 and SDM-Q-Doc were moderately correlated to relevant external criteria listed in our Table 5. Therefore, we tentatively concluded that both SDM-Q-9 and SDM-Q-Doc can be feasibly used for healthcare professionals to assess the degree of shared decision making among different types of cancer survivors.

As we compared our findings to previous studies on cancer survivors,^7,12,13^ slightly differences were found. Wu et al^12^ found that item 1 in the SDM-Q-9 was misfit for the breast cancer survivors, and they recommended removing this item from SDM-Q-9. In contrast, our findings indicate that item 1 fit in the SDM-Q-9; however, item 1 as compared with other items did perform worse in the unidimensionality. Specifically, both infit and outfit MnSq of item 1 were the highest among the MnSq of other items. That is, item 1 was deviated from the underlying concept of shared decision making in the SDM-Q-9; however, the deviation is strong and unacceptable in the findings from Wu et al^12^ but is weak and acceptable in our findings. Additionally, Calderon et al^13^ like Wu et al^12^ found that item 1 may jeopardize the unidimensional structure in the SDM-Q-9. Calderon et al^13^ found that the SDM-Q-9 could have one-factor structure if removing item 1; contrarily, the SDM-Q-9 fit better to a two-factor structure if item 1 retained in the questionnaire. A possible reason is the different samples: we recruited a sample with more heterogeneous diagnoses in cancers than did Wu et al^12^ and Calderon et al^13^ Given that our sample was more diverse than the samples of Wu et al^12^ and Calderon et al,^13^ we would recommend retaining item 1 in the SDM-Q-9. Nevertheless, the psychometric evidence of SDM-Q-9 is still insufficient, and future studies are warranted to examine whether item 1 should be retained.

Calderon et al^7^ recently studied the psychometric properties of the SDM-Q-Doc, and they found that the SDM-Q-Doc was significantly correlated to physician satisfactory. Our findings also demonstrated similar correlation between SDM-Q-Doc and physician satisfactory. However, the factorial structure of SDM-Q-Doc was found to be different between the findings of Calderon et al^7^ and those of ours. Specifically, Calderon et al^7^ used exploratory factor analysis and CFA to conclude a two-factor structure for the SDM-Q-Doc. In contrast, we used CFA and Rasch model to conclude a single-factor structure for the SDM-Q-Doc. We suspected that the number of recruited physicians could be a reason to cause such a difference. Calderon et al^7^ recruited 32 oncologists and we recruited a total of 154 physicians across three fields of expertise (gynecologist, oncologist, and urologist). Although the 32 oncologists in the study of Calderon et al^7^ rated the SDM-Q-Doc on 520 cancer survivors with different diagnoses, we can say that the two-factor structure of SDM-Q-Doc was solely based on the perspective of the 32 oncologists. On the other hand, the number of physicians in our study was much larger than that of Calderon et al^7^ Thus, the representativeness of our participants should be stronger than that of the participants in Calderon et al^7^ However, future studies are warranted to confirm the factorial structure of SDM-Q-Doc because to the best our knowledge, only Calderon et al^7^ and we have examined the factorial structure for SDM-Q-Doc on cancer patients.

In addition to the three studies on cancer patients,^7,12,13^ our psychometric results were in line with the studies on populations other than cancer patients. In the SDM-Q-9, Scholl et al^33^ found that it has high internal consistency (α = 0.92) with satisfactory corrected item-total correlations (0.52 to 0.85) among patients with type 2 diabetes, chronic pain, and depression. De las Cuevas et al,^34^ also found satisfactory internal consistency (α = 0.89) in the SDM-Q-9; they further concluded that the SDM-Q-9 has a unidimensional structure after removing item. In the SDM-Q-Doc, Simon et al^35^ like our results demonstrate the unidimensional structure derived from Rasch analysis.

### Strength and limitations

The study has the following strengths. First, a standard and robust translation process was applied to both SDM-Q-9 and SDM-Q-Doc to tackle their cultural adaptation, linguistic validity, and paralleled characteristics. Second, the sample in our study was heterogeneous and large. For cancer survivors, we recruited 1783 patients with different cancer diagnoses. For physicians, we recruited 154 experts in different fields of expertise. Therefore, the representativeness of our sample should be strong. Third, we applied comprehensive and thorough testing in psychometrics to examine both SDM-Q-9 and SDM-Q-Doc. With all the psychometric findings were satisfactory, the use of SDM-Q-9 and SDM-Q-Doc is highly recommended in clinical practice.

There are some limitations in this study. First, although our sample is large and heterogeneous, all the participants were recruited in Iran. Therefore, the use of SDM-Q-9 and SDM-Q-Doc is restricted for Iranian. Second, following by the first limitation, our findings cannot be generalized to cancer survivors who are in severe condition. Specifically, we only recruited those with apparently good cognitive ability and had no serious psychiatric disorders. Therefore, we were unsure whether the SDM-Q-9 and SDM-Q-Doc can be used for cancer survivors with sever condition. However, given that patients should provide their opinions in the model of shared decision making, we believe that this limitation should not be serious. Third, the SDM-Q-Doc was completed by physicians for their patients. Therefore, each physician rated about 12 SDM-Q-Doc and it is possible that the SDM-Q-Doc questionnaires rated by the same physician had dependency. However, we did not tackle this into our psychometric testing because we have checked the dependencies using intraclass correlation coefficient. Because the intraclass correlation coefficient was low (0.04), we decided not to use multilevel analysis to fulfill the parsimony principle in psychometric testing.

## Conclusion

This study validated the SDM-Q-9 for use among various types of cancer survivors and the SDM-Q-Doc for use among physicians with different areas of expertise on their cancer patients. Given the increasing number of cancer survivors and the importance of shared decision making in oncology setting, using both measures to evaluate the level of shared decision making can help healthcare professionals provide personalized care efficiently ana subsequently improved the treatment outcomes.

## Ethical approval

All procedures performed in the study involving human participants were in accordance with the ethical standards of the institutional and/or national research committee and with the 1964 Helsinki declaration and its later amendments or comparable ethical standards. The study was approved by the Research Ethical Committee of Qazvin University of Medical Sciences, Qazvin, Iran. Written informed consent was obtained from all subjects. Informed consent was obtained from all individual participants included in the study.

## Competing interests

The authors declare no conflicts of interest, both financial and non-financial, for this study.

## Funding

No fund was received to conduct this study.

## Authors’ contributions

BN and AHP collected the initial data. CYL, BN, CCL and AHP were involved in the conception of the study, performed the analyses, and drafted the manuscript. AB, VI, and MB were involved in the interpretation of the results from the analyses.

**Table 1:** Characteristics of the participants (n = 1783 for patients; n = 154 for physicians)

**Patient**	
Age (y), M (SD)	58.63 (12.83)
Sex (male), No. (%)	928 (52.05%)
Educational year, M (SD)	6.03 (2.96)
Marital status, No. (%)	
Single	286 (16.04%)
Married	1198 (67.19%)
Widowed/divorced	299 (16.77%)
Mini-mental state exam, M (SD)	25.12 (1.90)
Karnofsky Performance Scale Score, M (SD)	70.68 (12.81)
Diagnosis, No. (%)	
Gastrointestinal cancer	582 (32.64%)
Breast cancer	284 (15.92%)
Multiple myeloma	193 (10.82%)
Skin cancer	264 (14.81%)
Lung cancer	212 (11.89%)
Genitourinary cancer	150 (8.41%)
Others	98 (5.50%)
Time since diagnosis in years, M (SD)	2.87 (1.66)
**Physicians**	
Age (years), M (SD)	38.69 (10.34)
Sex (Male), No. (%)	121 (78.58%)
Number of years employed; M (SD)	9.62 (5.81)
Profession, No. (%)	
Gynecologist	63 (40.91%)
Oncologist	34 (22.08%)
Urologist	57 (37.01%)

**Table 2:** Psychometric properties of the 9-Item Shared Decision-Making Questionnaire (SDM-Q-9) and Shared Decision-Making Questionnaire–Physician version (SDM-Q-Doc) in item level

	**Analyses from classical test theory**								**Analyses from Rasch**							
	**Acceptance (completion rates in %)**		**Factor loading** ^a^		**Item-total correlation**		**Test-retest reliability** ^b^		**Infit MnSq**		**Outfit MnSq**		**Difficulty**		**DIF contrast across gender** ^cd^	
**Item**	**SDM-Q-9**	**SDM-Q-Doc**	**SDM-Q-9**	**SDM-Q-Doc**	**SDM-Q-9**	**SDM-Q-Doc**	**SDM-Q-9**	**SDM-Q-Doc**	**SDM-Q-9**	**SDM-Q-Doc**	**SDM-Q-9**	**SDM-Q-Doc**	**SDM-Q-9**	**SDM-Q-Doc**	**SDM-Q-9**	**SDM-Q-Doc**
1	98.2	99.1	0.83	0.65	0.62	0.49	0.78	0.73	1.23	0.98	1.12	1.06	-0.16	-0.37	-0.08	0.43
2	97.3	97.3	0.82	0.69	0.70	0.53	0.93	0.82	1.01	0.82	1.09	0.85	-0.14	-0.14	-0.03	0.18
3	99.3	98.6	0.84	0.64	0.69	0.41	0.88	0.80	0.93	0.95	1.08	0.88	0.13	-0.72	-0.06	-0.39
4	96.7	99.3	0.84	0.60	0.73	0.54	0.73	0.74	1.02	0.98	0.89	0.98	-0.40	-0.31	0.01	0.17
5	96.9	98.7	0.88	0.76	0.77	0.50	0.70	0.76	0.85	1.02	0.83	0.99	-0.27	-0.14	0.28	0.03
6	99.3	97.4	0.82	0.67	0.72	0.54	0.75	0.70	0.96	1.16	0.98	1.15	0.33	0.45	-0.22	-0.08
7	98.6	99.1	0.78	0.88	0.73	0.69	0.88	0.78	0.93	0.80	1.09	0.85	0.42	0.59	-0.05	-0.18
8	98.4	99.3	0.94	0.85	0.75	0.73	0.72	0.77	0.99	0.91	0.96	0.96	0.28	0.65	0.12	0.16
9	97.3	99.3	0.77	0.69	0.73	0.46	0.86	0.86	0.97	1.10	0.91	1.12	-0.18	0.11	0.10	-0.02

Abbreviations: MnSq, mean square error; DIF, differential item functioning. ^a^ Based on the first-order confirmatory factor analysis. ^b^ Using Pearson correlation. ^c^ DIF contrast >0.5 indicates substantial DIF. ^d^ DIF contrast across gender = Difficulty for females-Difficulty for males.

**Table 3:** Psychometric properties of the 9-Item Shared Decision-Making Questionnaire (SDM-Q-9) and Shared Decision-Making Questionnaire–Physician version (SDM-Q-Doc) in scale level

**Psychometric testing**	**SDM-Q-9**	**SDM-Q-Doc**	**Suggested cutoff**
Ceiling effects (%)	2.13	5.19	<20
Floor effects (%)	3.87	7.80	<20
Internal consistency (Cronbach’s α)	0.919	0.820	>0.7
Confirmatory factor analysis			
χ^2^ (*df*)	78.60 (27)*	33.58 (27) *	Non-significant
Comparative fit index	0.971	0.968	>0.9
Tucker-Lewis index	0.944	0.939	>0.9
Root-mean square error of approximation	0.070	0.078	<0.08
Standardized root mean square residual	0.050	0.052	<0.08
Average Variance Extracted	0.70	0.52	>0.5
Composite Reliability	0.95	0.91	>0.6
Standard error of measurement	2.89	2.64	The smaller the better
Item separation reliability from Rasch	0.92	0.84	>0.7
Item separation index from Rasch	3.47	2.31	>2
Person separation reliability from Rasch	0.84	0.79	>0.7
Person separation index from Rasch	2.28	2.92	>2
Test-retest reliability by Pearson correlation	0.85	0.81	>0.4

*P < 0.001.

**Table 4:** Measurement invariance across gender on the 9-Item Shared Decision-Making Questionnaire (SDM-Q-9) and Shared Decision-Making Questionnaire–Physician version (SDM-Q-Doc) through confirmatory factor analysis

**Model and comparisons**	**Fit indices**							
	**χ** ^ 2 ^ ** (** ***df*** **)**	**∆χ** ^ 2 ^ **(∆** ***df*** **)**	**CFI**	**∆CFI**	**SRMR**	**∆SRMR**	**RMSEA**	**∆RMSEA**
**SDM-Q-9**								
M1: Configural	133.35 (54)*		0.969		0.048		0.063	
M2: Plus all loadings constrained	147.61 (63)*		0.973		0.045		0.060	
M3: Plus all intercepts constrained	159.93 (72)*		0.978		0.043		0.058	
M2−M1		14.26 (9)		0.004		-0.003		-0.003
M3−M2		12.32 (9)		0.005		-0.001		-0.002
**SDM-Q-Doc**								
M1: Configural	153.21 (54)*		0.965		0.051		0.065	
M2: Plus all loadings constrained	167.36 (63)*		0.970		0.048		0.061	
M3: Plus all intercepts constrained^a^	181.19 (72)*		0.973		0.046		0.059	
M2−M1		14.15 (9)		-0.005		-0.003		-0.004
M3−M2		13.83 (9)		0.003		-0.002		-0.002

Abbreviations: CFI, comparative fit index; SRMR, standardized root mean square residual; RMSEA, root mean square error of approximation. * *P *< 0.05 ^a^ M1 = Model 1, a configural model; M2 = Model 2, a model based on M1 with all factor loadings constrained being equal across groups; M3 = Mode 3, a model based on M2 with all item intercepts constrained being equal across groups.

**Table 5:** Correlations between the 9-Item Shared Decision-Making Questionnaire (SDM-Q-9), Shared Decision-Making Questionnaire–Physician version (SDM-Q-Doc), and external criteria

	**SDM-Q-Doc**	**SDM-Q-9**
SDM-Q-9	0.41	-
SDM-Q-Doc	-	0.41
Depression^a^	-0.60	-0.55
Anxiety^a^	-0.66	-0.62
Family Decision-Making Self-Efficacy Scale	0.52	-
Illness education^b^	0.53	-
Assertiveness^b^	0.62	-
Mindful non-adherence^b^	0.40	-
Insomnia Severity Index	-0.58	-0.49
Epworth Sleepiness Scale	-0.60	-0.53
Physician satisfaction	0.41	0.67
Patient satisfaction^c^	0.69	0.33

^a^ Measured using Hospital Anxiety and Depression Scale. ^b^ Measured using Patient Self-Advocacy Scale. ^c^ Measured using the 7-item Short Assessment of Patient Satisfaction. All *P* values < 0.001.
